# Treatment of volar defects of the finger using dorsal digital–metacarpal flap versus free medial plantar artery flap: a comparative study

**DOI:** 10.1186/s12893-020-00994-3

**Published:** 2021-01-22

**Authors:** Quanzhe Liu, Wenlai Guo, Wenrui Qu, Xiaolan Ou, Rui Li, Heng Tian

**Affiliations:** Department of Hand Surgery, the Second Hospital of Jilin University, Changchun, 13000 Jilin China

**Keywords:** Soft-tissue defect, Volar surface of finger, MPAF, DDMF, Nerve injury

## Abstract

**Background:**

The treatment of defects on the volar surface of the finger has been scarcely reported, and its utility for digital resurfacing remains unclear. This study compared the outcomes of free medial plantar artery flap (MPAF) and dorsal digital–metacarpal flap (DDMF) in finger reconstruction.

**Methods:**

This retrospective cohort study included 24 patients with soft-tissue defects on the volar surface of the finger from March 2014 to March 2017. The patients were divided into two groups: the MPAF group and the DDMF group. The operation time, complications, such as flap necrosis, graft loss, infection, paresthesia, and donor-site morbidity, as well as two-point discrimination (2-PD) were carefully recorded. The Michigan Hand Outcomes Questionnaire was used for conduct follow-up assessment.

**Results:**

After more than 12 months of follow-up, the MPAF group had a longer operative time compared with DDMF group, but there was no significant difference between postoperative complications and 2-PD test result in patients without nerve injury. And in terms of overall function, Modified VSS score and 2-PD test (the patients with nerve injury), There were relatively obvious statistical differences, MPAF was superior to DDMF (p < 0.005).

**Conclusion:**

MPAF and DDMF are reliable for reconstruction of the volar surface of the finger; however, MPAF offers better functional outcomes and is associated with a lower incidence of postoperative complications.

## Background

Soft-tissue defects on the volar surface of the finger are highly common and frequently accompanied by exposed tendons, digital nerves and vessels, therefore flap reconstruction is often necessary [1]. “Replacement of like with like” has become a key principle for plastic surgery techniques used in soft-tissue defect coverage.

The volar surface of the hand is composed of highly specialized skin that has its own structural characteristics, with good sensitivity and ability to restore skin stability to resist friction; these characteristics must be considered in reconstruction. Previous studies have reported, methods such as skin graft, cross-finger flap, local flap transfer, and free flap [2–4]. Although these methods can meet wound coverage requirements; none are perfect.

Dorsal digital–metacarpal flap (DDMF) is a simple and readily available option for digital reconstruction [5, 6]. Following the first description of the dorsal metacarpal artery by Holevich [7], Foucher and Brauna improved the technique and designed a sensate island flap raised on the first dorsal metacarpal artery flap with its concomitant veins and a sensory branch of the superficial radial nerve in 1979 [8]. This technique gained popularity and was used for the reconstruction of soft-tissue defects of the hand because it enabled not only wound defect coverage and provided robust blood supply; but also had relatively fewer technical requirements.

Plantar skin is glabrous and thick, with solid anchorage to the deep structures, similar to palmar skin. Shanahan et al. [9] first reported the use of the medial plantar sensory flap for the treatment of heel defects in 1979. Subsequently, Oberlin et al. [10] demonstrated that it provided stable, pliable, durable, innervated, glabrous, and non-hairy skin. This is a relatively ideal supply area to the palmar area. Moreover, with significant advances in microsurgery, it is not technically challenging to design free flaps based on the medial plantar artery.

Although some methods have been used to resurface whole palm defects and thumb contractures, the number of cases remains small, and the utility of these methods for digital resurfacing remains unclear. The purpose of this study was to compare the outcomes of free medial plantar artery flap (MPAF) and DDMF for finger reconstruction to provide better treatment suggestions.

## Methods

This retrospective cohort study adhered to the principles of the Helsinki declaration and was approved by Jilin University, China (license number: SCXK (Ji) 20140084). Patients with soft-tissue defects on the volar surface of the finger caused by trauma from March 2014 to March 2017 were included. All patients’ medical history was recorded. Detailed clinical examination and radiography were performed to assess the skeletal effects and arterial duplex scanning was performed to assess the vascular pattern of the hand.

Those who had systemic diseases, including diabetes mellitus, vascular sclerosis, and peripheral vascular disease, those who could not withstand lengthy operation, and those who had a smoking history were excluded from this study, along with those with accompanied fractures or tendon ruptures. Patients were informed about the merits and demerits of each method and made the choice themselves. Written informed consent was obtained from all patients.

A total of 28 patients were included, of these 4 (3 DDMF and 1 MPAF) were lost to follow-up and 24 were eventually included in the study with an average age of 38.9 years (20–65 years); Eighteen patients were men (75%), and six were women (25%). All skin and soft-tissue defects were caused by trauma. The defects were localized at the volar surface of the finger with exposed flexor tendons. The patients were divided into two groups: MPAF group and DDMF group, and all procedures were performed by a single surgeon. The locations of the defects were as follows: thumb, seven; index finger, four; middle finger, four; and ring finger, nine. Among the patients, 13 had only skin and soft-tissue defects, and the other 11 (MPAF: 6 cases; DDMF: 5 cases) had nerve defects/injury. Nerve repair was performed using different methods (MPAF: nerve bridging, DDMF: flap with neurovascular bundle or the proper digital dorsal nerve was anastomosed with the digital nerve). Demographic data of the patients is presented in Table 1.

**Table 1:** General characteristics of the patients

Variable	MPAF Group (N = 12)	DDMF Group (N = 12)	P Value
Age(y)	38.4 ± 2.4	39.2 ± 3.8	NS
Sex			NS
Male	8	10	
Female	4	2	
Defect site			NS
Thumb	3	4	
Index finger	2	2	
Middle finger	2	2	
Ring finger	5	4	
Nerve defect	N = 6	N = 5	NS
Thumb	0	0	
Index finger	1	1	
Middle finger	1	1	
Ring finger	4	3	
Defect size (cm^2^)	3.9 ± 1.9	3.8 ± 1.7	> 0.005

Data presented as mean ± standard error of the mean or n

*MPAF*: medial plantar artery flap, *DDMF* dorsal digital–metacarpal flap, *NS* not significant

A survey was undertaken at least 12 months after the surgery to investigate and record the operation time and complications, including flap necrosis, graft loss, infection, paresthesia, and donor-site morbidity. The Michigan Hand Outcomes Questionnaire (MHQ) [11] was used to assess patient satisfaction with the surgical result. The questionnaire consists of five domains, including hand function, activities of daily living, work performance, esthetics, and satisfaction with hand function and was scored on a scale from 0–to 100 (0 = worst result, 100 = best result). In particular, the esthetic appearance also measured with Modified Vancouver Scar Scale (VSS), the total score was 18, including pigmentation (0–3), vascularity (0–3), pliability (0–5), Height (0–3), Pain (0–2) and Prutitus (0–2) [12–14]. Differences between independent parametric variables were assessed using an independent samples t-test. The group were also divided two subgroups with and without nerve damage and the 2PD test [15, 16] always started from a greater distance between the spikes of the discriminator and then the distance was gradually reduced, generally from 10 mm to the smallest distance. The smallest distance the patient recognized as a sensation of two points was recorded in millimeters and used for analysis.

The overall function was graded as excellent, good, or poor. We defined the criteria for excellent results as flap survival and mean MHQ score of ≥ 85 without complications; flap survival with mean MHQ score of 60–84 and minimum complications were the criteria for good results. A result was considered poor when an alternative reconstructive procedure was required or the mean MHQ score was < 60.

### Surgical method/technique

The wound was subjected to radical debridement before reconstruction because most cases were a result of trauma. Necrotic tissue was removed, and antibiotic therapy was administered on the basis of microbiology results until the local wound bacterial culture confirmed the absence of infection. Thereafter, flap and nerve bridging were performed to repair the soft-tissue and nerve defects in the second stage.

The operation was performed under general anesthesia or nerve block. The pneumatic tourniquet was applied to provide a bloodless field. Surface marker measurement and preoperative imaging were performed; antibiotics were injected intravenously before tourniquet application.

**Fig. 1:**
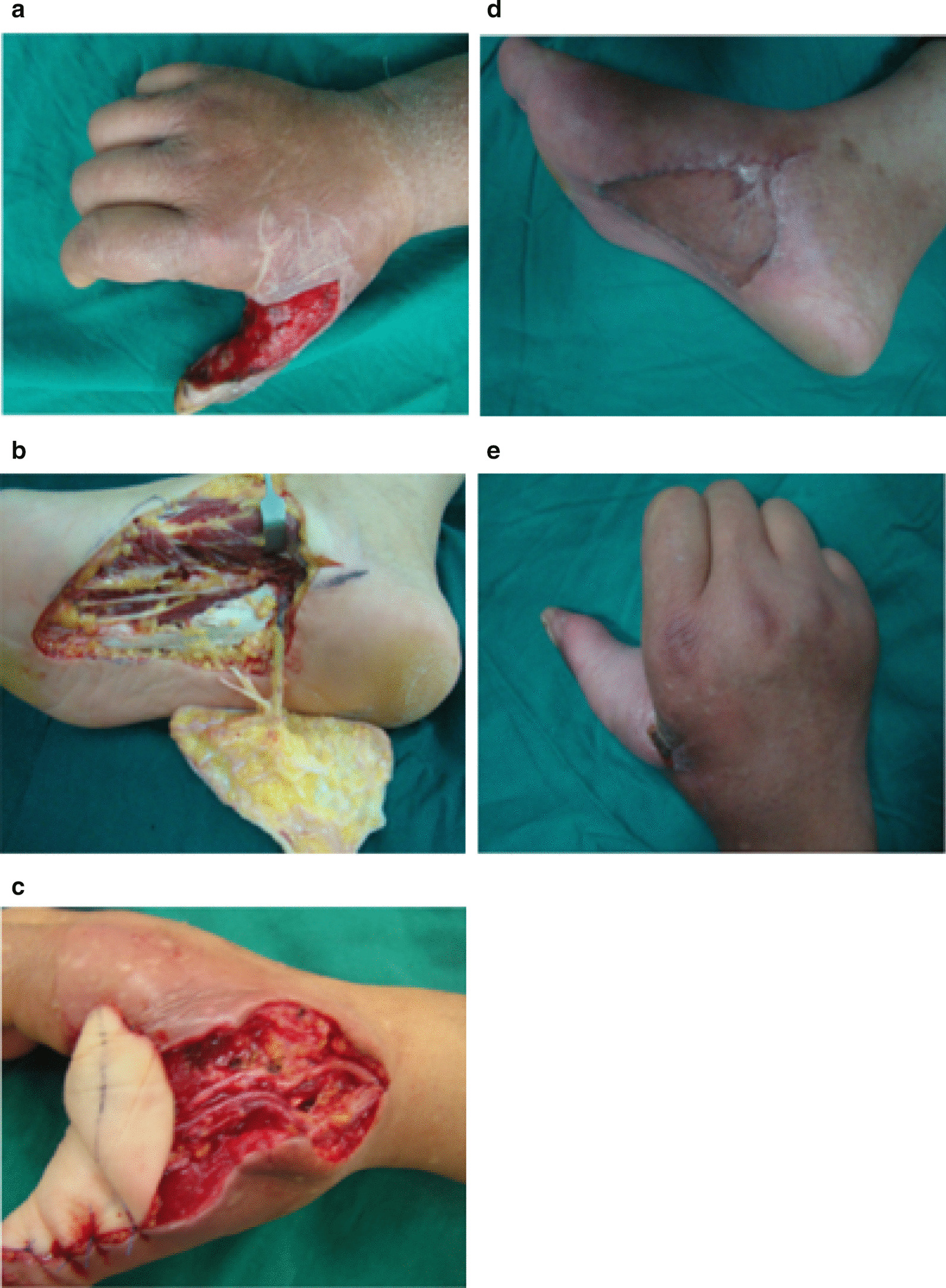
MPAF method. **a** A 45-year-old man with post-traumatic soft-tissue defect of the thumb after debridement. **b** Harvesting of MPAF. **c** Intraoperative image showing neurovascular anastomosis. **d**, **e** Postoperative image showing wound healing of the flap and skin graft

MPAF is located in the non-weight-bearing area of the plantar on both sides of the axis and behind the head of the metatarsal bone. The size and shape of the flap can be designed and dissected as per the wound size, but it generally cannot be more than 4 × 8 cm [17, 18]. The medial plantar artery and t medial plantar nerve can be identified between the abductor hallucis and flexor digitorum brevis. The flap was then elevated at the superficial muscle membrane of the abductor hallucis and isolated in the distal to the proximal direction. The medial plantar artery was anastomosed with the digital proper arteries or the common palmar digital arteries; the dorsal veins of the finger or palm were anastomosed with the accompanying vein of the plantar metatarsal artery. To ensure that the flap was sensate, the branches supplying the flap were isolated and teased out from the main trunk of the medial plantar nerve. The proximal and distal ends of the flap nerve should be sutured with the proper digital nerve. The donor site of the flaps was primarily grafted with a split-thickness skin graft Fig. 1.

**Fig. 2:**
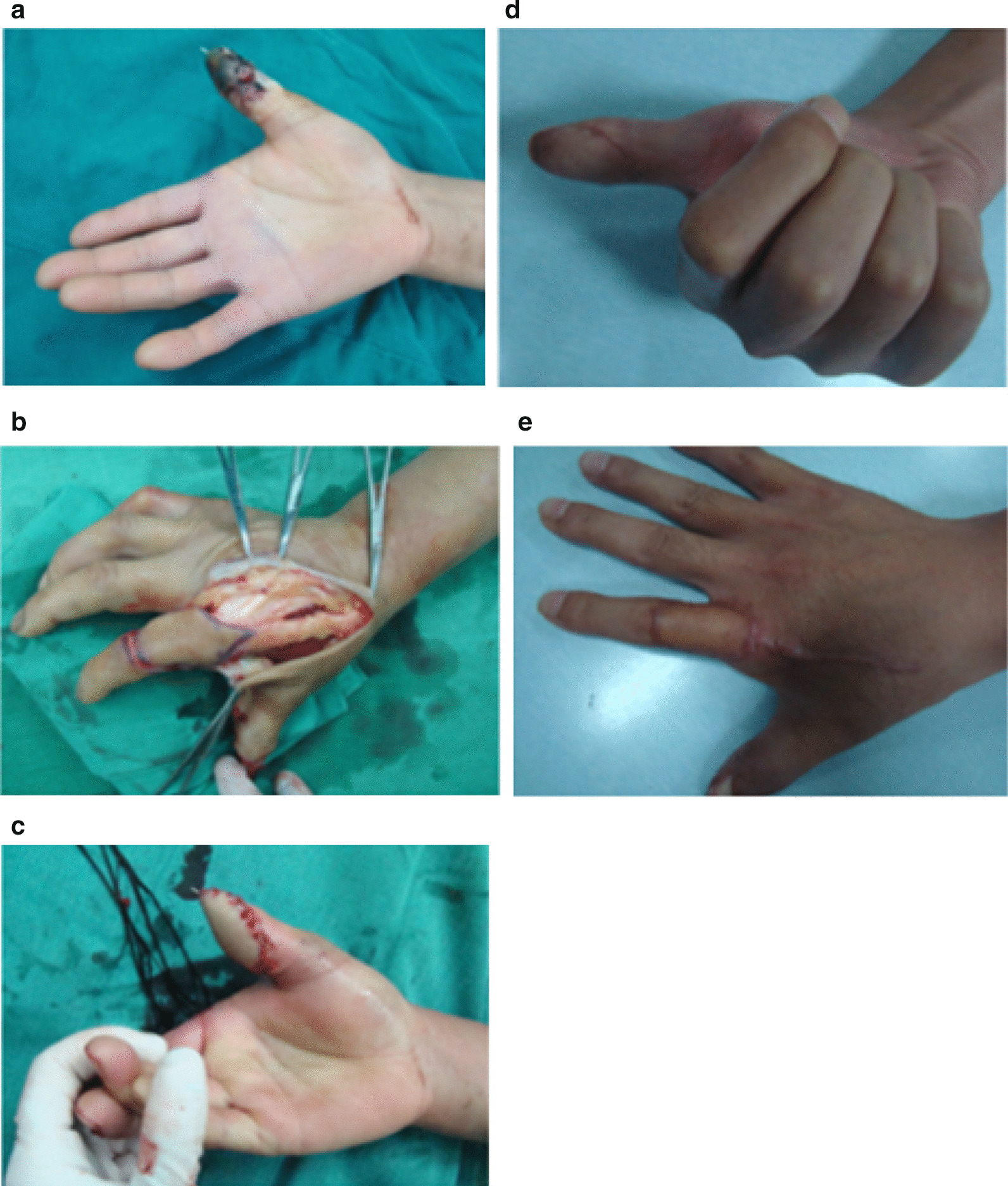
DDAF method. **a** A 42-year-old man with post-traumatic necrosis of the distal segment of the right thumb. **b** Harvesting of DDAF. **c**, **d** The wound was covered immediately, and the donor site was covered with a split-thickness skin graft. **e** Postoperative image showing wound healing of the flap and skin graft

DDMF was located between the metacarpals and with the rotation point located at the proximal phalanx level. It was designed on the intermetacarpal spaces as an ellipse centered over the dorsal metacarpal arteries that were ligated at the proximal margin of the flap. The flap was elevated in the interosseous fascial plane. The pivot point of the flap was located at the mid-point of the proximal phalanx where the proximal dorsal branch of the digital artery anastomoses with the dorsal digital artery. It can be transferred to the defect through an open tunnel, and the secondary defect was closed primarily or with a skin graft Fig. 2.

### Statistical analyses

Data are presented as the mean ± standard error of the mean. The incidence of complications, functional outcomes, and other qualitative parameters were compared using Fisher’s exact test. The mean operation time between the two groups and other quantitative variables were analyzed using the t-test. The level of significance was set at p < 0.05.

## Results

The follow-up period was 12–18 months (mean 14.6 months). There were no significant differences between the groups in the terms of age, sex, etiology, nerve injury, or site and size of the defect. Despite infection in five patients (three MPAF and two DDMF), none developed necrotic flaps after dressing changes and required anti-infection treatment.

In all patients, the donor site had no serious complications, such as skin necrosis and graft loss, except for a relatively unsightly scar; however, all patients exhibited an uneventful recovery course and good graft outcome. Even those in the MPAF group healed well or had callus formation, and experienced no adverse effect on walking. However, one patient in the DDMF group had a complication of hyperplastic scar contracture that decreased the range of motion; it improved with rehabilitation.

Complete weight bearing and hospital stay time were significantly earlier and shorter in the DDMF (3–5 days) group than in the MPAF (9–12 days) group (p < 0.05). The defect size was comparable in both groups (p > 0.05) (Table 2). The largest flap was 2 × 4 cm, and the smallest flap was 2 × 1 cm. There was a significant difference in the operation time; the MPAF group had a significantly longer operation time than the DDMF group (117 ± 5.0 vs. 73 ± 3.9 min; p < 0.001).

**Table 2:** Outcomes

Variable	MPAF group, (N = 12)	DDMF group, (N = 12)	Normal finger	p value
Operative time (min)	117 ± 5.0	73 ± 3.9		< 0.001
Weight-bearing time (days)	10 ± 0.94	4.0 ± 0.74		< 0.001
2PD				
With nerve injury(mm)	4.55 ± 0.27 (n = 6)	4.7 ± 0.20 (n = 7)		< 0.001
Without nerve injury (mm) p value	4.55 ± 0.27 (n = 6) p < 0.001	4.7 ± 0.20 (n = 7) p = 0.001		= 0.439
VSS score	1.79 ± 1.5	3.15 ± 2.9	0	< 0.001
MHQ score	86.48 ± 2.52	78.22 ± 3.06	100	< 0.005
Overall function				0.001
Excellent	10 (83.3)	1 (8.33)		
Good	2 (16.7)	11 (91.67)		
Poor	0	0		

Data presented as mean ± standard error of the mean

2-PD: two-point discrimination. MHQ: Michigan Hand Outcomes Questionnaire VSS: Vancouver Scar Scale

There was no significant difference in the two-point discrimination (2-PD) test results in the patients without nerve injury between the two groups (p = 0.414), both of which were better than the nerve injured cases (p < 0.05). However, there were obvious differences among the patients with nerve injury; the MPAF results were superior to the DDMF results (p = 0.004).

The esthetics (modified VSS score) and MHQ results were included in the evaluation of overall functional recovery. After more than 6 months of follow-up, the esthetic appearance of the MPAF group was closer to normal. In addition, the MPAF group had a higher VSS score than the DDMF group (p < 0.001).

In the MPAF group, 10 patients showed excellent results, and 2 showed good results. In the DDMF group, 1 patient showed excellent result and 11 showed good results. The improvement in the functional outcome was greater in the MPAF group than in the DDMF group (p < 0.005).

## Discussion

Hand trauma accounts for approximately 12% of all trauma cases and is the most common reason for emergency treatment, accounting for approximately one-fifth of all emergency patients [19, 20]. The results of defect coverage are closely associated with the patient's quality of life, functional recovery, and appearance. The difficulty lies in following the principle of “like with like”. Both the appearance and functionality need to be considered.

The volar surface of the finger has a particular structure and characteristics, such as thickness and toughness with poor mobility, poor flexibility, thick cuticle layer, and absence of hair follicles. Although the development of flap surgery has led to the development of several repair methods, the number of cases limited and the utility of these methods for digital resurfacing is yet to be fully clarified. Currently, DDMF [21–23] and free MPAF [22, 24, 32] commonly used. To determine which method is superior and provides better treatment outcomes, we compared the outcomes of these two methods (DDMF and MPAF) to evaluate the effects.

According to the anatomy of the finger and the defect size, Morrison and Yang found that MPAF can be designed and dissected less to than 4 × 8 cm [17, 18]. Backhach et al. [25] and Pelissier et al. [26] also reported that DDMF with a large donor site can allow total coverage of digital skin defects. Thus, both DDMF and MPAF can resolve moderate-size defects. In the present study, the largest defect was 2 × 4 cm, and the smallest was 2 × 1 cm. There was no significant difference between the two groups (p > 0.05).

The operation time was significantly longer in the MPAF group than in the DDMF group (117 ± 5.0 vs. 73 ± 3.9 min; p < 0.001), both of which were performed by a single surgeon. In a similar comparative study, Wael Hussein Mahmoud [27] found that the operation time was longer for medial plantar flap than for distally based sural artery flap (90–130 vs. 60–100 min). This could be explained by the dissection of the medial plantar flap and micro-technique that make the process difficult [28, 29]. Although microsurgery is commonly performed in clinical practice following advances in technology and equipment, it has the limitation of being tedious and technically demanding. In contrast, DDMF with neurovascular bundle offers reliable blood supply and is relatively simpler in terms of technique without vascular anastomosis.

In our series, recovery of sensation in the flap was measured using a static 2-PD test. On an average, there was no significant difference between the uninjured patients in the two groups (p = 0.414), both of which were better than the injured patients (p < 0.05). However, there were obvious differences between the injured patients in the two groups; the results of MPAF were superior to those of DDMF (p = 0.004). Similarly, Yang reported that DDMF as a retrograde flap is not sensitive, except in rare cases in which the proper digital dorsal nerve was anastomosed with the digital nerve [30, 31]. However, unlike in our study, Qi-Shun Huang reported no significant difference between bridging cases and uninjured cases, and both were better than those that were sutured no tension when using MPAF to repair volar skin defects [32].

Donor-site morbidity following harvest of the flap is another issue that should be considered while evaluating the overall functional effect. We found that the donor site defect can be closed directly in DMAF leaving only one linear scar on the dorsum of the hand. In contrast, the donor site defect needed a skin graft for resolution in DDAF and MPAF. Hyperpigmentation and hypertrophic scarring are common complications [33] after full-thickness skin grafting that creates a gross mismatch in color and affects metacarpophalangeal joint motion. The esthetic result is potentially inferior in ethnic groups with darker pigmented skin. However, the donor site of MPAF is located on the non-weight-bearing position of the foot in contrast to that for DDAF which has the obvious advantage of concealment without affecting the function of the foot. According to the follow-up questionnaire, patient satisfaction was significantly better with MPAF than with DDMF, especially among young female patients, even if only one linear scar was left with DMAF. Moreover, if the flap is oversized and extends proximally over the metacarpophalangeal joint, it may decrease the range of motion because of hyperplastic scar contracture. In our series, there was one case with a similar situation; the patient showed improvement following rehabilitation. However, there was no dysfunction in MPAF except local pigmentation, which is consistent with previous reports by relevant scholars [32].

The limitations of the study include the small sample size, lack of random treatment allocation, and biases associated with the surgeon assessing the outcomes. Thus, larger, prospective, randomized, blinded studies are required to better ascertain the efficacy of the outcomes.

## Conclusions

Both MPAF and the DDMF are available for reconstruction of the volar surface of the finger. Irrespective of its technical requirements, DDMF is a single-stage procedure and has a wide arc of flap transposition, which prevents the need of a free flap [34]. MPAF is an ideal donor site and can resolve nerve defects. Although postoperative recovery of hand function mainly depends on trauma, other conditions were excluded, and the repair of skin and soft-tissue and nerve defects were compared between these two techniques. We found that MPAF offers better functional outcomes with a lower incidence of postoperative complications.

## Data Availability

All data generated or analyzed during this study are included in this article and is available from the corresponding author upon reasonable request.

## Abbreviations

MPAF: Medial plantar artery flap
DDMF: Dorsal digital–metacarpal flap
2-PD: Two-point discrimination
MHQ: Michigan Hand Outcomes Questionnaire
VSS: Vancouver Scar Scale

## References

[1] Zaksar K, Toros T, Sugun TS, Bal E, Ademoglu Y, Kaplan I (2009). Reconstruction of finger pulp defects using homodigital dorsal middle phalangeal neurovascular advancement flap. J Hand Surg..

[2] Okazaki M, Hasegawa H, Kano M (2005). A different method of fingertip reconstruction with the thenar flap. Plast Reconstr Surg..

[3] Cohen BE, Cronin ED (1983). An innervated cross-finger flap for fingertip reconstruction. Plast Reconstr Surg..

[4] Yang JW, Kim JS, Lee DC (2010). The radial artery superficial palmar branch flap: a modified free thenar flap with constant innervation. J Reconstr Microsurg..

[5] Muyldermans T, Hierner R (2009). First dorsal metacarpal artery flap for thumb reconstruction: a retrospective clinical study. Strateg Trauma Limb Reconstr..

[6] Chen C, Zhang X, Shao X (2010). Treatment of thumb tip degloving injury using the modified first dorsal metacarpal artery flap. J Hand Surg Am..

[7] Holevich J (1963). A new method of restoring sensibility to the thumb. J Bone Joint Surg Br.

[8] Foucher G, Braun JB (1979). A new island flap transfer from the dorsum of the index to the thumb. Plast Reconstr Surg..

[9] Shanahn RE, Gingrass RP (1979). Medial plantar sensory flap for coverage of heel defects. Plast Reconstr Surg..

[10] Oberlin C, Saffar P (1984). The internal plantar island flap. Anatomic study and surgical applications. Rev Chir Orthop Reparatrice Appar Mot..

[11] Chung KC, Pillsbury MS, Walters MR, Hayward RA (1988). Reliability and validity testing of the Michigan Hand Outcomes Questionnaire. J Hand Surg Am..

[12] Oliveira GV, Chinkes D, Mitchell C, Oliveras G, Hawkins HK, Herndon DN (2005). Objective assessment of burn scar vascularity, erythema, pliability, thickness, and planimetry. Dermatol Surg..

[13] Baryza MJ, Baryza GA (1995). The Vancouver Scar Scale: an administration tool and its interrater reliability. J Burn Care Rehabil..

[14] Yeong EK, Mann R, Engrav LH (1997). Improved burn scar assessment with use of a new scar-rating scale. J Burn Care Rehabil..

[15] Bardak AN, Alp M, Erhan B, Paker N, Kaya B, Önal AE (2009). Evaluation of the clinical efficacy of conservative treatment in the management of carpal tunnel. Adv Therapy..

[16] Akalin E, El O, Peker O, Senocak O, Tamci S, Gülbahar S, Cakmur R, Oncel S (2002). Treatment of carpal tunnel syndrome with nerve and tendon gliding exercises. Am J Phys Med Rehabil..

[17] Morrison WA, Crabb DM, O’Brien BM, Jenkins A (1983). The instep of the foot fascio cutaneous island and as a free flap for heel defects. Plast Reconstr Surg..

[18] Yang KM, Xu DC, Shi J, Li ZH (2001). Applied anatomy of free bifolicated flap based on the cutaneous branch of medial plantar superficial artery. Chin J Clin Anat.

[19] Niska R, Bhuiya F, Xu J (2010). National hospital ambulatory medical care survey: 2007 emergency department summary. Natl Health Stat Report.

[20] Maroukis BL, Chung KC, Mac EM (2016). Hand trauma care in the United States: a literature review. Plast Reconstru Surg..

[21] Muyldermans T, Hierner R (2009). First dorsal metacarpal artery flap for thumb reconstruction: a retrospective clinical study. Strategies Trauma Limb Reconstr..

[22] Chen C, Zhang X, Shao X (2010). Treatment of thumb tip degloving injury using the modified first dorsal metacarpal artery flap. J Hand Surg Am..

[23] Chang SC, Chen SL, Chen TM (2004). Sensate first dorsal metacarpalartery flap for resurfacing extensive pulp defects of the thumb. Ann Plast Surg..

[24] Sen SK, O'Connor EF, Tare M (2015). The free instep flap for palmar and digital resurfacing. J Plast Reconstr Aesthet Surg..

[25] Bakhach J, Demiri E, Conde A, Baudet J (1999). Lelambeauméta-carpien dorsal à pédiculerétrogradeétendu, étudeanatomique et àpropos de 22 cascliniques. Ann Chir Plast Esthet.

[26] Pelissier P, Casoli V, Bakhach J, Martin D, Baudet J (1999). Reverse dorsal digital and metacarpal Xaps: a review of 27 cases. Plast Reconstr Surg..

[27] Mahmoud WH (2017). Foot and ankle reconstruction using the distally based sural artery flap versus the medial plantar flap: a comparative study. J Foot Ankle Surg, May-Jun.

[28] Akyurek M, Safak T, Sonmez E, Ozkan O, Kecik A (2004). A new flap design: neral-island flap. Plast Reconstr Surg..

[29] Kim KS, Kim ES, Kim DY, Lee SY, Cho BH (2003). Resurfacing of totally degloved hand using thin perforator-based cutaneous free flaps. Ann Plast Surg..

[30] Yang D, Morris SF (2001). Reversed dorsal digital and metacarpal island flaps supplied by the dorsal cutaneous branches of the palmar digital artery. Ann Plast Surg..

[31] Yang D, Morris SF (2001). Vascular basis of dorsal digital and metacarpal skin flaps. J Hand Surg..

[32] Huang QS, Wu X, Zheng HY (2015). Medial plantar flap to repair defects of palm volar skin. Eur J Trauma Emerg Surg..

[33] Jaquet Y, Enepekides DJ, Torgerson C, Higgins KM (2012). Radial forearm free flap donor site morbidity: ulnar-based transposition flap vs split-thickness skin graft. Arch Otolaryngol Head Neck Surg..

[34] Yan H, Ouyang Y, Chi Z, Gao W, Zhang F, Fan C (2012). Digital pulp reconstruction with free neurovascular toe flaps. Aesthetic Plast Surg..

